# *catena*-Poly[[chlorido(methyl phenyl sulfide-κ*S*)mercury(II)]-μ-chlorido]

**DOI:** 10.1107/S1600536808013718

**Published:** 2008-05-14

**Authors:** Ludmila Vigo, Pekka Salin, Raija Oilunkaniemi, Risto S. Laitinen

**Affiliations:** a Department of Chemistry, PO Box 3000, FI-90014 University of Oulu, Finland

## Abstract

The title compound, [HgCl_2_(C_7_H_8_S)]_*n*_, was isolated from the reaction of MeSPh with HgCl_2_. The Hg^II^ atom has a distorted tetra­hedral geometry and is coordinated by one S atom and three Cl atoms. Two of the Cl atoms act as bridging ligands between the Hg atoms, forming a two-dimensional polymeric structure.

## Related literature

For related literature, see: Peindy *et al.* (2005 ▶). 
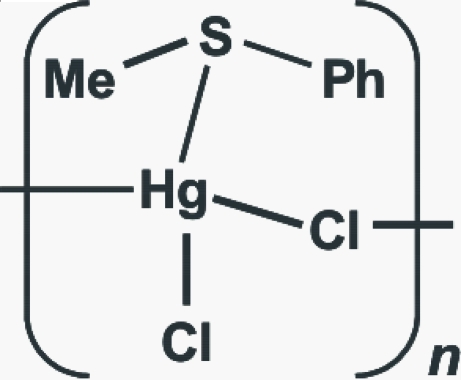


## Experimental

### 

#### Crystal data

[HgCl_2_(C_7_H_8_S)]
*M* *_r_* = 395.68
Orthorhombic, 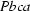

*a* = 5.9616 (12) Å
*b* = 14.935 (3) Å
*c* = 22.142 (4) Å
*V* = 1971.4 (7) Å^3^
*Z* = 8
Mo *K*α radiation
μ = 16.30 mm^−1^
*T* = 150 (2) K
0.25 × 0.10 × 0.08 mm

#### Data collection

Bruker–Nonius KappaCCD diffractometer
Absorption correction: multi-scan (*SHELXTL*; Sheldrick 2008 ▶) *T*_min_ = 0.106, *T*_max_ = 0.355 (expected range = 0.081–0.271)
9374 measured reflections
1760 independent reflections
1562 reflections with *I* > 2σ(*I*)
*R*_int_ = 0.063

#### Refinement

*R*[*F* ^2^ > 2σ(*F* ^2^)] = 0.035
*wR*(*F* ^2^) = 0.092
*S* = 1.07
1760 reflections
102 parameters
H-atom parameters constrained
Δρ_max_ = 2.22 e Å^−3^
Δρ_min_ = −1.51 e Å^−3^

### 

Data collection: *COLLECT* (Nonius, 1998 ▶); cell refinement: *DENZO-SMN* (Otwinowski & Minor, 1997 ▶); data reduction: *DENZO-SMN*; program(s) used to solve structure: *SHELXS97* (Sheldrick, 2008 ▶); program(s) used to refine structure: *SHELXL97* (Sheldrick, 2008 ▶); molecular graphics: *DIAMOND* (Brandenburg & Berndt, 1999 ▶); software used to prepare material for publication: *WinGX* (Farrugia, 1999 ▶).

## Supplementary Material

Crystal structure: contains datablocks I, global. DOI: 10.1107/S1600536808013718/sg2244sup1.cif

Structure factors: contains datablocks I. DOI: 10.1107/S1600536808013718/sg2244Isup2.hkl

Additional supplementary materials:  crystallographic information; 3D view; checkCIF report

## Figures and Tables

**Table 1:** Selected bond lengths (Å)

Hg1—Cl1	2.3429 (18)
Hg1—S1	2.4548 (17)
Hg1—Cl2	2.6050 (17)
Hg1—Cl2^i^	2.742 (2)

Symmetry code: (i) 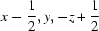.

**Fig. 1.**
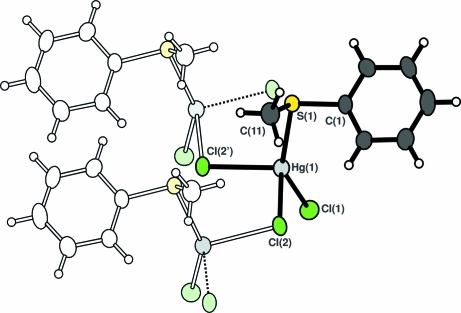
The molecular structure of I indicating the numbering of the atoms. The thermal ellipsoids have been drawn at 50% probability.

**Fig. 2.**
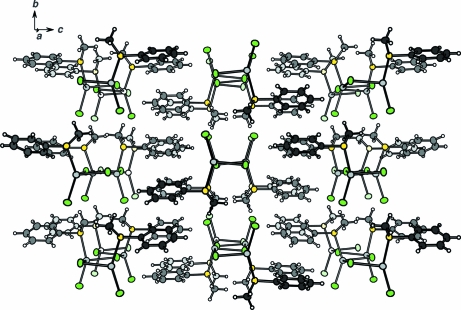
The packing of polymer chains.

## supplementary crystallographic information

### Comment

Crystals of [HgCl_2_(MeSPh)]_n_ (I) were isolated from the reaction of MeSPh with HgCl_2_ in EtOH. The asymmetric unit of I consists of one Hg atom, MeSPh ligand and two chlorine atoms. The mercury(II) atom has distorted tetrahedral geometry and is coordinated to one sulfur atom and three chlorine atoms. Two of the chlorine atoms act as bridging ligands between the mercury atoms forming a two-dimensional polymeric structure. The Hg - Cl bond lenghts are 2.6050 (17) and 2.742 (2) Å for the bridging chlorines and 2.3429 (18) Å for the terminal chlorine, The Hg - S bond length is 2.4548 (17) Å. The bond parameters can be compared to those in [{PhS(CH_2_)SPh}Hg_2_Cl_4_]_n_ where Hg atom has a similar coordination environment (Peindy *et al.* (2005)]

### Experimental

The addition of MeSPh (0.603 g; 4.85 mmol) to HgCl_2_ (0.283 g: 1.04 mmol) in 10 ml EtOH gave at first a clear solution followed by precipitation of colourless crystals. Decomposition of the crystals took place upon removal of the solvent. Crystals suitable for crystal structure determination were picked from the reaction solution.

### Refinement

H atoms were positioned geometrically and refined using a riding model with C—H = 0.95 - 0.98 Å and with *U*_iso_(H) = 1.2 *U*_eq_(C).

### Crystal data

**Table:**

[HgCl_2_(C_7_H_8_S)]	*F*_000_ = 1440
*M**_r_* = 395.68	*D*_x_ = 2.666 Mg m^−^^3^
Orthorhombic, *P**b**c**a*	Mo *K*α radiation λ = 0.71073 Å
Hall symbol: -P 2ac 2ab	Cell parameters from 1562 reflections
*a* = 5.9616 (12) Å	θ = 3.7–25.4º
*b* = 14.935 (3) Å	µ = 16.30 mm^−^^1^
*c* = 22.142 (4) Å	*T* = 150 (2) K
*V* = 1971.4 (7) Å^3^	Needle, colourless
*Z* = 8	0.25 × 0.10 × 0.08 mm

### Data collection

**Table:**

Bruker–Nonius KappaCCD diffractometer	1760 independent reflections
Radiation source: fine-focus sealed tube	1562 reflections with *I* > 2σ(*I*)
Monochromator: graphite	*R*_int_ = 0.063
*T* = 150(2) K	θ_max_ = 25.5º
φ scans, and ω scans with κ offsets	θ_min_ = 3.7º
Absorption correction: multi-scan(SHELXTL; Sheldrick 2008)	*h* = −7→7
*T*_min_ = 0.106, *T*_max_ = 0.355	*k* = −18→16
9374 measured reflections	*l* = −23→26

### Refinement

**Table:**

Refinement on *F*^2^	Hydrogen site location: inferred from neighbouring sites
Least-squares matrix: full	H-atom parameters constrained
*R*[*F*^2^ > 2σ(*F*^2^)] = 0.035	*w* = 1/[σ^2^(*F*_o_^2^) + (0.0473*P*)^2^ + 8.371*P*] where *P* = (*F*_o_^2^ + 2*F*_c_^2^)/3
*wR*(*F*^2^) = 0.092	(Δ/σ)_max_ < 0.001
*S* = 1.07	Δρ_max_ = 2.22 e Å^−^^3^
1760 reflections	Δρ_min_ = −1.51 e Å^−^^3^
102 parameters	Extinction correction: SHELXL97 (Sheldrick, 2008), Fc^*^=kFc[1+0.001xFc^2^λ^3^/sin(2θ)]^-1/4^
Primary atom site location: structure-invariant direct methods	Extinction coefficient: 0.0014 (3)
Secondary atom site location: difference Fourier map	

### Special details

**Table:**

Geometry. All e.s.d.'s (except the e.s.d. in the dihedral angle between two l.s. planes) are estimated using the full covariance matrix. The cell e.s.d.'s are taken into account individually in the estimation of e.s.d.'s in distances, angles and torsion angles; correlations between e.s.d.'s in cell parameters are only used when they are defined by crystal symmetry. An approximate (isotropic) treatment of cell e.s.d.'s is used for estimating e.s.d.'s involving l.s. planes.
Refinement. Refinement of *F*^2^ against ALL reflections. The weighted *R*-factor *wR* and goodness of fit *S* are based on *F*^2^, conventional *R*-factors *R* are based on *F*, with *F* set to zero for negative *F*^2^. The threshold expression of *F*^2^ > σ(*F*^2^) is used only for calculating *R*-factors(gt) *etc*. and is not relevant to the choice of reflections for refinement. *R*-factors based on *F*^2^ are statistically about twice as large as those based on *F*, and *R*- factors based on ALL data will be even larger.

### Fractional atomic coordinates and isotropic or equivalent isotropic displacement parameters (Å^2^)

**Table:**

	*x*	*y*	*z*	*U*_iso_*/*U*_eq_	
Hg1	0.04378 (5)	0.284473 (18)	0.188615 (12)	0.03063 (19)	
Cl1	0.0758 (3)	0.43434 (12)	0.15855 (8)	0.0366 (4)	
Cl2	0.0860 (3)	0.26914 (14)	0.30526 (7)	0.0327 (4)	
S1	0.1319 (3)	0.13004 (11)	0.15897 (7)	0.0284 (4)	
C1	0.0764 (12)	0.1286 (5)	0.0799 (3)	0.0282 (15)	
C2	−0.1212 (14)	0.1616 (5)	0.0562 (3)	0.0353 (16)	
H2	−0.2335	0.1856	0.0820	0.042*	
C3	−0.1532 (14)	0.1592 (5)	−0.0055 (3)	0.0405 (18)	
H3	−0.2899	0.1802	−0.0223	0.049*	
C4	0.0143 (15)	0.1260 (6)	−0.0432 (4)	0.045 (2)	
H4	−0.0072	0.1256	−0.0857	0.054*	
C5	0.2088 (15)	0.0941 (5)	−0.0191 (3)	0.0417 (19)	
H5	0.3216	0.0711	−0.0451	0.050*	
C6	0.2443 (13)	0.0948 (4)	0.0425 (3)	0.0343 (15)	
H6	0.3804	0.0728	0.0591	0.041*	
C7	−0.0891 (15)	0.0613 (6)	0.1887 (3)	0.0371 (18)	
H11A	−0.2345	0.0881	0.1787	0.056*	
H11B	−0.0737	0.0570	0.2327	0.056*	
H11C	−0.0796	0.0013	0.1709	0.056*	

### Atomic displacement parameters (Å^2^)

**Table:**

	*U*^11^	*U*^22^	*U*^33^	*U*^12^	*U*^13^	*U*^23^
Hg1	0.0363 (3)	0.0265 (2)	0.0291 (2)	−0.00098 (11)	−0.00083 (10)	−0.00019 (10)
Cl1	0.0428 (10)	0.0268 (9)	0.0401 (10)	−0.0015 (7)	0.0006 (8)	0.0041 (7)
Cl2	0.0319 (8)	0.0446 (10)	0.0217 (8)	0.0000 (8)	−0.0009 (6)	0.0017 (7)
S1	0.0325 (9)	0.0279 (8)	0.0247 (8)	0.0024 (7)	−0.0007 (7)	−0.0011 (6)
C1	0.035 (4)	0.028 (3)	0.021 (3)	−0.003 (3)	0.001 (3)	0.002 (3)
C2	0.048 (4)	0.028 (4)	0.030 (4)	0.005 (3)	−0.002 (3)	−0.004 (3)
C3	0.052 (5)	0.038 (4)	0.031 (4)	−0.003 (4)	−0.003 (3)	0.000 (3)
C4	0.067 (5)	0.042 (4)	0.024 (4)	−0.013 (4)	−0.003 (4)	0.001 (3)
C5	0.058 (5)	0.039 (4)	0.029 (4)	−0.002 (4)	0.009 (4)	−0.004 (3)
C6	0.043 (4)	0.028 (3)	0.033 (4)	0.002 (3)	0.006 (3)	−0.002 (3)
C7	0.046 (4)	0.030 (4)	0.036 (4)	−0.011 (4)	0.002 (3)	0.003 (3)

### Geometric parameters (Å, °)

**Table:**

Hg1—Cl1	2.3429 (18)	C3—C4	1.392 (12)
Hg1—S1	2.4548 (17)	C3—H3	0.9500
Hg1—Cl2	2.6050 (17)	C4—C5	1.362 (12)
Hg1—Cl2^i^	2.742 (2)	C4—H4	0.9500
Cl2—Hg1^ii^	2.742 (2)	C5—C6	1.381 (10)
S1—C1	1.782 (7)	C5—H5	0.9500
S1—C7	1.795 (8)	C6—H6	0.9500
C1—C2	1.381 (10)	C7—H11A	0.9800
C1—C6	1.393 (10)	C7—H11B	0.9800
C2—C3	1.380 (10)	C7—H11C	0.9800
C2—H2	0.9500		
			
Cl1—Hg1—S1	143.53 (7)	C2—C3—H3	119.9
Cl1—Hg1—Cl2	110.97 (7)	C4—C3—H3	119.9
S1—Hg1—Cl2	99.31 (6)	C5—C4—C3	120.1 (7)
Cl1—Hg1—Cl2^i^	100.08 (6)	C5—C4—H4	120.0
S1—Hg1—Cl2^i^	98.49 (6)	C3—C4—H4	120.0
Cl2—Hg1—Cl2^i^	92.28 (5)	C4—C5—C6	120.9 (7)
Hg1—Cl2—Hg1^ii^	97.92 (6)	C4—C5—H5	119.5
C1—S1—C7	102.5 (3)	C6—C5—H5	119.5
C1—S1—Hg1	103.6 (2)	C5—C6—C1	118.6 (7)
C7—S1—Hg1	106.4 (3)	C5—C6—H6	120.7
C2—C1—C6	121.1 (6)	C1—C6—H6	120.7
C2—C1—S1	121.8 (5)	S1—C7—H11A	109.5
C6—C1—S1	117.0 (5)	S1—C7—H11B	109.5
C3—C2—C1	119.0 (7)	H11A—C7—H11B	109.5
C3—C2—H2	120.5	S1—C7—H11C	109.5
C1—C2—H2	120.5	H11A—C7—H11C	109.5
C2—C3—C4	120.2 (8)	H11B—C7—H11C	109.5
			
Cl1—Hg1—Cl2—Hg1^ii^	−77.75 (8)	C7—S1—C1—C6	−120.4 (6)
S1—Hg1—Cl2—Hg1^ii^	81.56 (7)	Hg1—S1—C1—C6	129.1 (5)
Cl2^i^—Hg1—Cl2—Hg1^ii^	−179.453 (9)	C6—C1—C2—C3	1.3 (11)
Cl1—Hg1—S1—C1	−40.3 (3)	S1—C1—C2—C3	179.5 (6)
Cl2—Hg1—S1—C1	173.5 (2)	C1—C2—C3—C4	−1.6 (11)
Cl2^i^—Hg1—S1—C1	79.7 (2)	C2—C3—C4—C5	1.3 (12)
Cl1—Hg1—S1—C7	−147.9 (3)	C3—C4—C5—C6	−0.6 (12)
Cl2—Hg1—S1—C7	65.8 (3)	C4—C5—C6—C1	0.3 (11)
Cl2^i^—Hg1—S1—C7	−28.0 (3)	C2—C1—C6—C5	−0.6 (10)
C7—S1—C1—C2	61.4 (7)	S1—C1—C6—C5	−178.9 (5)
Hg1—S1—C1—C2	−49.2 (6)		

Symmetry codes: (i) *x*−1/2, *y*, −*z*+1/2; (ii) *x*+1/2, *y*, −*z*+1/2.
